# Integrated transcriptomic and proteomic analysis reveals inflammatory activation and blood-brain barrier disruption during meningitis-associated extraintestinal pathogenic *Escherichia coli* infection

**DOI:** 10.1080/21505594.2026.2670939

**Published:** 2026-05-11

**Authors:** Zhiwei Li, Meili Chen, Yangyang Du, Kaixiang Jia, Yi Lu, Xiaoying Yu, Xuefeng Cao, Lianci Peng, Rendong Fang

**Affiliations:** a Joint International Research Laboratory of Animal Health and Animal Food Safety, College of Veterinary Medicine, Southwest University, Chongqing, China; b National Center of Technology Innovation for Pigs, Chongqing, China; c Division of Infectious Diseases and Geographic Medicine, Department of Medicine, Stanford University, Stanford, CA, USA

**Keywords:** Bacterial meningitis, blood-brain barrier, *Escherichia coli*, transcriptomics, proteomics

## Abstract

Meningitis-associated extraintestinal pathogenic *Escherichia coli* (ExPEC) is a major cause of bacterial meningitis, yet the molecular mechanisms underlying blood-brain barrier (BBB) disruption during infection remain unclear. We employed integrated transcriptomic and proteomic analysis to investigate host responses of human cerebral microvascular endothelial cell line hCMEC/D3 to ExPEC strain RS218 infection. Multi-omics integration revealed coordinated immune activation, with upregulation of innate immune signaling pathways such as Toll-like receptor, NOD-like receptor, TNF, and IL-1 signaling, as well as antigen presentation pathways. In addition, we identified direct molecular evidence for BBB compromise, including concordant downregulation of tight junction protein ZO-1 at both transcriptomic and proteomic levels, validated by immunofluorescence showing reduced ZO-1 expression in infected cells. Several processes that may contribute to BBB breakdown were identified, such as glycosaminoglycan degradation, cytoskeletal reorganization, and suppression of TGF-β/SMAD signaling. Moreover, extensive metabolic dysregulation was evident, including downregulation of neural metabolic support functions and compromised protein homeostasis. Abundant discordance between transcriptomic and proteomic levels revealed complex post-transcriptional control mechanisms. *In vitro* experiments demonstrated RS218-induced cell death in brain endothelial cells, microglial cells, and peritoneal macrophages. Animal experiments confirmed systemic metabolic disruption, immune cell alteration, functional BBB disruption, and profound brain cytokine elevation. This integrated analysis advances our understanding of bacterial meningitis pathogenesis and identifies potential therapeutic targets.

## Introduction

Central nervous system (CNS) infections caused by bacteria present major clinical challenges globally [1]. These infections often lead to serious complications including brain damage and death, even when patients receive appropriate treatment [2]. The meningitis-associated extraintestinal pathogenic *Escherichia coli* (ExPEC) is an important pathogen in this context since it can cause meningitis in newborns and immunocompromised individuals [3]. Unlike other major meningitis pathogens such as *Streptococcus pneumoniae*, *Neisseria meningitidis*, and *Haemophilus influenzae*, no effective vaccines are currently available for meningitis-associated ExPEC infections [1,4].

The blood-brain barrier (BBB) constitutes a highly selective cellular interface that maintains CNS homeostasis by regulating the exchange of molecules between the systemic circulation and brain tissue [5]. This barrier consists of specialized endothelial cells that line brain blood vessels, connected by tight junctions that control what can pass through. Important proteins in these junctions include Claudin-5, Occludin, and ZO-1 [6]. Astrocytes and pericytes also contribute to barrier function [6]. When this barrier is damaged, bacteria and inflammatory molecules can enter the brain and cause serious problems [7].

The BBB acts as the first line of defense against circulating pathogens [6]. However, meningitis-causing bacteria have evolved specialized mechanisms to breach this protective barrier, leading to CNS invasion and subsequent neuroinflammation [8]. Meningitis pathogens cross the BBB mainly through three classical mechanisms: the paracellular route via disruption of intercellular tight junctions, the transcellular pathway involving intracellular trafficking, and the Trojan horse mechanism in which infected immune cells serve as vehicles [9]. Beyond these classical invasion routes, bacterial pathogens trigger severe CNS inflammatory responses and release cytotoxic factors that enhance BBB permeability. The production of pro-inflammatory cytokines including IL-1β, IL-6, and TNF-α is principally responsible for BBB breakdown during neuroinflammation [10], while bacterial toxins and virulence factors directly cause cellular damage and disrupt barrier function [8,11].

Meningitis-associated ExPEC breaches the BBB through coordinated virulence factors and host signaling manipulation [8]. Key virulence factors include IbeA protein, which binds to Caspr1 receptors on brain microvascular endothelial cells and activates focal adhesion kinase signaling to promote bacterial internalization [12]. The α-hemolysin (HlyA) disrupts astrocyte-endothelial communication by inhibiting signaling mediated by TGFBRII and Gli1/2 [13]. Additionally, the outer membrane protein OmpA activates protein kinase C-α in brain endothelial cells, leading to β-catenin dissociation from vascular endothelial cadherin, thereby increasing BBB permeability [14].

From the host perspective, meningitis-associated ExPEC infection triggers multiple signaling cascades that compromise BBB integrity. The infection upregulates vascularization factors including VEGFA [15], PDGF-B [16], and ANGPTL4 [17], which disrupt BBB structure through different mechanisms. Furthermore, infection activates transcription factors like Snail-1 that negatively regulate junction proteins [15]. Additionally, infection induces expression of regulatory non-coding RNAs including circ_2858 [18] and lncRSPH9-4 [19], which modulate VEGFA and MMP3 expression, thereby exacerbating tight junction degradation. Recently, meningitis-associated ExPEC-induced endothelial cell pyroptosis has emerged as a potential mechanism contributing to BBB disruption [20].

Previous studies have typically focused on individual pathways or molecules separately, lacking a systematic perspective on the complex host-pathogen interactions during bacterial meningitis. Better understanding how bacteria affect the BBB requires a comprehensive approach. The human cerebral microvascular endothelial cell line hCMEC/D3 has emerged as a well-established *in vitro* model for studying BBB dysfunction, providing insights into the molecular mechanisms underlying pathogen-host interactions [21].

Here, we performed integrated RNA sequencing and data-independent acquisition (DIA) proteomic analyses to characterize the host response of hCMEC/D3 cells to RS218 infection. By comparing changes at both mRNA and protein levels, we aimed to identify which responses are coordinated and which show differences that might indicate post-transcriptional regulation. This integrated approach provides insights into how meningitis-associated ExPEC disrupts the BBB integrity and identifies potential therapeutic targets.

## Materials and methods

### Bacterial strains and culture conditions

The meningitis-associated extraintestinal pathogenic *E. coli* (ExPEC) strain RS218 was obtained as a generous gift from Professor Xiangru Wang at the College of Veterinary Medicine, Huazhong Agricultural University, Wuhan, China. Bacterial stocks were routinely cultured in Luria-Bertani (LB) liquid medium (Qingdao Hope Bio-Technology Co., Ltd., China) at 37 °C with continuous shaking until reaching exponential growth phase. For bacterial counting and colony selection, cultures were plated on LB agar plates (Qingdao Hope Bio-Technology Co., Ltd., China) and incubated at 37 °C for 12 hours.

### Cell culture

Human cerebral microvascular endothelial cells (hCMEC/D3) were obtained from Jennio Biotech (Guangzhou Jennio Biotech Co., Ltd., China) and served as the *in vitro* blood-brain barrier (BBB) model. The cells were maintained in Dulbecco’s Modified Eagle Medium (DMEM; Gibco, USA) enriched with 10% fetal bovine serum (FBS; ExCell Bio, China) under controlled culture conditions of 37 °C and 5% CO_2_.

### Bacterial infection

The hCMEC/D3 cells were challenged with RS218 strain at a multiplicity of infection (MOI) of 1 as previously described with modifications [20]. Briefly, cells were seeded into 12-well plates and cultured for 24 hours until reaching appropriate confluence. Before infection, cells were washed three times with serum-free medium, then incubated with bacterial suspension (100 μL bacteria +400 μL medium per well) at 37 °C with 5% CO_2_. The infection duration was modified based on downstream analysis requirements. For transcriptomic analysis, cells were exposed to bacterial infection for 2 hours, while for proteomic analysis, the infection duration was extended to 3 hours. This was to capture robust protein-level changes that typically require longer timeframes than transcriptional responses. Mock groups were processed identically but without bacterial exposure. Following the designated infection periods, cells were harvested for subsequent RNA extraction and protein extraction procedures.

### RNA sequencing

Total RNA was extracted from infected and mock-treated hCMEC/D3 cells using the TRIzol reagent (Accurate Biotechnology, China). The RNA integrity number (RIN) was measured by an Agilent Bioanalyzer 2100 system (Agilent Technologies, USA). Only RNA samples meeting quality control standards were used for library construction. Paired-end libraries were prepared using the ABclonal mRNA-seq Lib Prep Kit (ABclonal, China). PCR products were purified and library quality was assessed on the Agilent Bioanalyzer 2100 system. Sequencing was performed with an Illumina NovaSeq 6000 instrument. Analyses of the data generated from the Illumina platform were performed using an in-house pipeline from Shanghai Applied Protein Technology, China. Raw sequencing data in FASTQ format were initially processed using in-house Perl scripts to remove adapter sequences and filter out low-quality reads, obtaining clean reads for subsequent analysis. Clean reads were aligned to the reference genome using HISAT2 software [22]. FeatureCounts [23] was used to calculate the read counts mapped to each gene, and FPKM values were calculated based on gene length.

### Transcriptomic bioinformatics analysis

Differential expression analysis was performed using DESeq2 [24] to compare RS218-infected cells versus mock controls. The criteria for differentially expressed genes (DEGs) were padj < 0.05 and absolute log2 fold changes (LFC) >1. Volcano plots were generated using VolcaNoseR [25]. Principal component analysis (PCA) was performed using ExpressAnalyst [26].

For heatmap visualization, the top 100 DEGs were selected based on two criteria: (1) the lowest padj values and (2) the highest absolute LFC. Heatmaps were generated using TBtools [27] and row-scaled for visualization. Functional enrichment analysis was conducted using ExpressAnalyst [26] with the DEGs. Six pathway databases were utilized for comprehensive functional annotation: KEGG pathway, Reactome pathway, Gene Ontology Biological Process (GO:BP), Gene Ontology Molecular Function (GO:MF), Gene Ontology Cellular Component (GO:CC), and binding motif analysis. The top 10 enriched terms from each category were displayed using ridgeline plots, where color intensity represents statistical significance (*p* value) and distribution shapes reflect directional regulation patterns.

For protein-protein interaction (PPI) network analysis, upregulated and downregulated DEGs were analyzed separately using the STRING database [28]. Cytoscape [29] was used to visualize the PPI networks and to identify hub genes. The top 30 hub genes were identified using the Maximal Clique Centrality (MCC) algorithm in the cytoHubba plugin [30]. Functional enrichment analysis of hub genes was performed using the built-in enrichment tool of STRING [28], focusing on Gene Ontology Biological Process (GO:BP) terms. The RNA-seq data for this study have been deposited in the European Nucleotide Archive (ENA) at EMBL-EBI under accession number PRJEB94539.

### Protein extraction and mass spectrometry

Protein was extracted from infected and mock-treated hCMEC/D3 cells using SDT lysis buffer (4% SDS, 100 mM Tris-HCl, pH 7.6). Protein concentration was determined using the BCA assay. All samples were processed using the Filter Aided Proteome Preparation (FASP) method for trypsin digestion [31]. Briefly, dithiothreitol (DTT) was added to each sample to a final concentration of 40 mM and incubated at 600 rpm for 1.5 hours at 37 °C for protein reduction. After cooling to room temperature, iodoacetamide (IAA) was added to a final concentration of 20 mM to alkylate reduced cysteine residues and incubated for 30 minutes in darkness. Samples were then transferred to ultrafiltration units (Microcon, 10 kDa MWCO) and washed three times with 100 μL UA buffer (8 M urea in 100 mM Tris-HCl, pH 8.5), followed by two washes with 100 μL 25 mM NH_4_HCO_3_ buffer. Trypsin was added at a trypsin-to-protein ratio of 1:50 (w/w) and incubated at 37 °C for 15–18 hours. After enzymatic digestion, peptides were desalted using C18 cartridges, lyophilized, and resuspended in 40 μL of 0.1% formic acid solution. Peptide concentration was determined by OD_280_ measurement and iRT standard peptides were added to each sample before mass spectrometry analysis.

Data-independent acquisition (DIA) mass spectrometry analysis was performed using the Thermo Scientific Orbitrap Astral mass spectrometer coupled with a Vanquish Neo UHPLC system (Thermo Scientific, USA). The mass spectrometer was operated in positive ion mode with a precursor ion scan range of 380–980 m/z. MS1 scans were acquired at a resolution of 240,000 at 200 m/z, with a normalized AGC target of 500% and a maximum injection time (IT) of 5 ms. MS2 data were acquired in DIA mode, using 299 isolation windows (2 m/z), with an HCD collision energy of 25 eV, normalized AGC target of 500%, and maximum IT of 3 ms.

### Proteomic bioinformatics analysis

DIA data were processed using DIA-NN software. The analysis parameters were set as follows: trypsin was specified as the digestion enzyme, allowing a maximum of one missed cleavage. Carbamidomethylation of cysteine (C) was set as a fixed modification, while oxidation of methionine (M) and acetylation of the protein N-terminus were specified as variable modifications. Protein identifications were filtered at a false discovery rate (FDR) of <1%. Protein quantification was based on fragment ion intensities and performed using the DIA-NN software. Statistical analysis was performed using t-test to calculate *p* values. Differentially expressed proteins (DEPs, RS218-infected cells versus mock controls) were defined by a *p* value < 0.05 and an absolute LFC > 0.585. Consistent presence/absence proteins were identified based on detection in >50% of samples in one group while being absent in all samples of the other group. These proteins were excluded from volcano plot analysis but included in subsequent functional enrichment analyses.

Proteomic bioinformatics analysis followed similar approaches to those described for transcriptomic analysis, with the following modifications. Volcano plots were generated excluding consistent presence/absence proteins. Principal component analysis included both DEPs and consistent presence/absence proteins. For heatmap visualization, the top 100 DEPs were selected based on *p* values, with an additional separate heatmap generated for consistent presence/absence proteins. Functional enrichment analysis and PPI network analysis included both DEPs and consistent presence/absence proteins. For enrichment analysis, proteins present in RS218-infected cells but absent in mock cells were assigned the maximum upregulated fold change observed in the dataset, while proteins absent in RS218-infected cells but present in mock cells were assigned the maximum downregulated fold change observed in the dataset. For functional enrichment analysis of DEPs, KEGG pathway, Reactome pathway, PANTHER Biological Process (PANTHER:BP), PANTHER Molecular Function (PANTHER:MF), PANTHER Cellular Component (PANTHER:CC), and binding motif analysis were utilized. For hub protein functional enrichment analysis, Reactome pathway terms were used. The mass spectrometry proteomics data have been deposited to the ProteomeXchange Consortium via the PRIDE [32] partner repository with the dataset identifier PXD066627.

### Integrative transcriptomic and proteomic analysis

For integrative analysis between transcriptomic and proteomic datasets, DEGs were defined using padj < 0.05 and DEPs were defined using *p* value < 0.05, without fold change thresholds to capture broader regulatory patterns. All analyses included consistent presence/absence proteins. Upregulated and downregulated DEGs and DEPs were analyzed separately.

Venn diagrams were generated using TBtools [27] to identify overlapping genes between transcriptomic and proteomic datasets. For concordantly regulated genes, heatmaps were constructed using the R package ComplexHeatmap [33], displaying the top 30 shared genes selected by transcriptomic padj values with z-score normalized expression levels.

Functional enrichment analysis was performed using Metascape [34] by importing DEGs and DEPs as multiple gene lists. Bubble plots displaying shared enriched terms were generated using SRplot [35]. For discordant regulation analysis, genes showing opposite regulation patterns between transcriptomic and proteomic levels were identified using Venn diagrams in TBtools [27] and functional enrichment analysis was performed using Metascape [34] with results visualized using SRplot [35].

### Cell death assay

The hCMEC/D3 cells were infected with RS218 at MOI = 1 for 3 hours. Cytotoxicity was assessed by measuring lactate dehydrogenase (LDH) release using the LDH Cytotoxicity Assay Kit (Beyotime, China) according to the manufacturer’s instructions. For inhibitor experiments, hCMEC/D3 cells were pretreated for 1 h with BAPTA-AM (10 μM), Necrostatin-1 (10 μM), VX-765 (10 μM), or Z-DEVD-FMK (20 μM) (all from MedChemExpress, USA) prior to RS218 infection. For BV2 cells and murine peritoneal macrophages (PMs), RS218 was applied at MOI = 0.1, 1, 10, and 100 for 1 h and 2 h.

### Immunofluorescence staining of RS218-infected cells

The hCMEC/D3 cells were infected with RS218 at MOI = 1 for 3 hours, then fixed with 4% paraformaldehyde and blocked with 5% BSA. Cells were incubated with primary antibody against ZO-1 (21773–1-AP, Proteintech, 1:2000) overnight at 4 °C, followed by Alexa Fluor 488-conjugated secondary antibody (ab150077, Abcam, 1:500) for 1 hour at room temperature in the dark, and DAPI counterstaining for 5 minutes. Images were acquired using a confocal microscope (FV3000, Olympus) and analyzed with ImageJ 1.52a. Relative ZO-1 positive area and fluorescence intensity were calculated with mock control set as 100%, and all ZO-1 measurements were normalized to corresponding DAPI intensities to correct for cell density differences.

### Animal study

C57BL/6 mice were purchased from the Chongqing Academy of Chinese Materia Medica (Chongqing, China) and maintained under Specific Pathogen-Free (SPF) conditions. All experiments were conducted using 8–10 week-old mice and were approved by the Institutional Animal Care and Use Committee (IACUC) of Southwest University, Chongqing, China (IACUC-20221022-08). All animal experiments in this study were in accordance with the ARRIVE guidelines.

For biochemical, hematological, and cytokine analyses, 15 mice were randomly assigned to three groups: mock control, 4-hour infection, and 12-hour infection groups. Infection groups received 2 × 10^7^ colony-forming units (CFU)/100 μL RS218 inoculum via tail vein injection, while mock controls received equivalent PBS. Blood samples were collected at 4 and 12 hours post-infection under isoflurane anesthesia for biochemical and hematological analysis. The mice were then euthanized by CO_2_ inhalation followed by cervical dislocation, and brain tissues were additionally harvested from the 12-hour group for cytokine measurement. Brain tissues were homogenized in ice-cold PBS containing protease inhibitor cocktail using an MP FastPrep-24 homogenizer (6.0 m/s, 60 seconds, twice). The homogenates were centrifuged at 10,000×*g* for 10 minutes at 4 °C to remove debris and cell aggregates, and the supernatants were collected and stored at −80 °C until analysis. Cytokine levels were quantified using the MILLIPLEX® Mouse Cytokine Expansion Panel (MCYT1-190K, MilliporeSigma, USA) according to manufacturer’s instructions. Briefly, 25 µL of brain tissue supernatant was added to each well containing 25 µL of antibody-immobilized magnetic beads. After overnight incubation at 2–8 °C with agitation on a plate shaker, plates were washed three times with wash buffer. Then 25 µL of detection antibodies were added and incubated for 1 hour at room temperature, followed by addition of 25 µL streptavidin-phycoerythrin for 30 minutes. After three washes, beads were resuspended in 150 µL sheath fluid and analyzed on Luminex 200 Instrument System.

For Evans Blue extravasation analysis, mice were challenged with RS218 as described above and 200 μL of 0.5% Evans Blue solution was injected via tail vein 30 min before the end of infection. Mice were then perfused via the left ventricle with PBS containing heparin sodium (10 U/mL), followed by 4% paraformaldehyde. Brain tissues were collected and Evans Blue extravasation was quantified by gray intensity analysis using ImageJ 1.52a. For histological analysis, after perfusion, brain tissues were fixed in 4% paraformaldehyde and processed for hematoxylin and eosin (H&E) staining. For western blot analysis, brain tissues collected at 4 and 8 hours post-infection were homogenized in RIPA lysis buffer supplemented with 1 mM PMSF, centrifuged at 10,000×*g* for 15 min at 4 °C, and supernatants were collected for SDS-PAGE and western blot analysis using antibodies against NLRP3 (Proteintech, 19,771–1-AP), Caspase-1 (ABclonal, A18646), Caspase-11 (AdipoGen, AG-20T-0140-C100), Claudin-5 (Proteintech, 29,767–1-AP), and β-Actin (Beyotime, AA128).

### Statistical analysis

Unless otherwise specified, all statistical analyses were performed using GraphPad Prism 10.1.0 (GraphPad Software, USA). For comparisons among three or more groups with a single variable, one-way analysis of variance (ANOVA) followed by Dunnett’s multiple comparisons test was used. For comparisons involving two independent variables, two-way ANOVA followed by Dunnett’s multiple comparisons test was used. For pairwise comparisons between two groups, unpaired Student’s t-test was applied. Statistical significance was set at *p* < 0.05. Significance levels are indicated as follows: *p < 0.05, **p < 0.01, ***p < 0.001, and ****p < 0.0001.

## Results

### Overview of transcriptomic and proteomic differential expression analysis

Transcriptomic analysis identified 738 differentially expressed genes (DEGs) in RS218 compared to Mock conditions, with 458 genes upregulated and 280 genes downregulated (Figure S1A, Table S1). Principal component analysis based on these DEGs showed clear separation between the two conditions, with PC1 explaining 99.7% of the variance (Figure S1B).

Proteomic analysis revealed 325 differentially expressed proteins (DEPs) in RS218 compared to Mock, including 175 upregulated and 150 downregulated proteins (Figure S1C, Table S2). Additionally, 39 proteins were specifically detected in RS218-infected cells but absent in Mock cells, while 47 proteins showed the opposite pattern (Table S2). These consistent presence/absence proteins were excluded from the volcano plot but included in PCA. PCA based on all DEPs effectively separated the two conditions, with PC1 explaining 90.8% of the variance (Figure S1D).

### Differentially expressed genes indicate inflammatory activation and cellular dysfunction following RS218 infection

**Figure 1.**
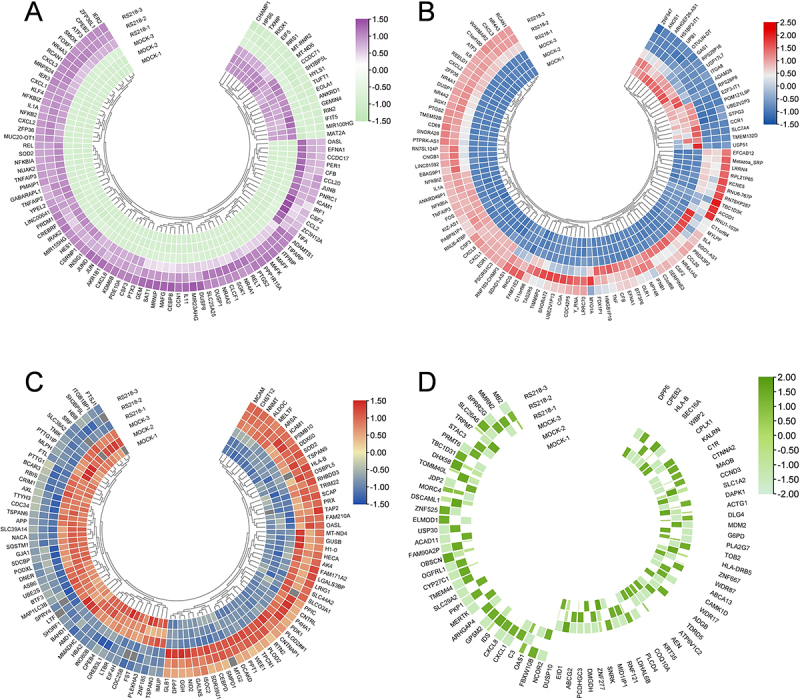
Representative differentially expressed genes and proteins between RS218 and mock conditions.

The top 100 DEGs selected by padj values showed predominant upregulation caused by RS218, with activation of inflammatory genes including transcription factors (NFKB2, REL, JUN, JUNB, JUND, CEBPB), pro-inflammatory cytokines and chemokines (IL1A, IL11, CXCL1, CXCL2, CXCL3, CXCL8, CCL2, CCL20, CSF2, CSF3), and key inflammatory mediators (PTGS2, ICAM1, TIFA, TNFAIP2) (Figure 1(A)). The top 100 DEGs selected by absolute LFC also demonstrated predominant upregulation caused by RS218, with several inflammatory mediators overlapping with the padj-selected set, including IL1A, CXCL1, CXCL2, CXCL3, CXCL8, CCL20, CSF2, CSF3, and PTGS2 (Figure 1(B)). This LFC-based selection identified additional pro-inflammatory cytokines such as TNF, IL6, and IFNB1, and immune activation marker CD69, reflecting a robust inflammatory response to RS218 infection.

(A) Heatmap showing abundance of representative differentially expressed genes (DEGs) selected based on the lowest padj values. (B) Heatmap showing abundance of representative DEGs selected based on the highest absolute log2 fold changes (LFC). (C) Heatmap showing abundance of representative differentially expressed proteins (DEPs) selected based on the lowest *p* values. (D) Heatmap showing presence/absence proteins between RS218 and Mock conditions. All heatmaps are row-scaled for visualization.

Differently, the top 100 DEPs selected by *p* values showed approximately equal numbers of upregulated and downregulated proteins (Figure 1(C)). Upregulated proteins included immune factors CEBPD, TRIM22, DDX60, OASL, TAP2, ICAM1, LGALS3BP, and MCAM. Downregulated proteins included junction molecules (GJA1), extracellular matrix interaction regulators (SDCBP, ITGB1BP1), barrier transport proteins (SLC38A2), neural cell surface proteins (APP), iron transport proteins (LTF, FTL), and cell cycle regulators (CDC25B, CDC34, PTTG1), suggesting compromised endothelial barrier integrity and cellular homeostasis. OASL, ICAM1, and SOD2 were consistently upregulated in both transcriptomic and proteomic top 100 datasets, while SH3BP5L was downregulated in both analyses (Figure 1(A,C)).

Proteins detected in RS218-infected cells but absent in Mock cells included antiviral response proteins (OAS1, DHX58), complement system components (C3), and inflammatory mediators (CXCL1, CXCL8), with CXCL1 and CXCL8 being identified across multiple analyses (Figure 1(A–D)). Conversely, proteins detected in Mock cells but absent in RS218-infected cells included synaptic function-related proteins (PCDHGC3, CTNNA2, DLG4, CPLX1) and cell cycle regulators (CCND3, MDM2), reflecting compromised neuronal connectivity and cellular homeostasis (Figure 1(D)).

### Transcriptomic enrichment analysis reveals inflammatory activation and novel regulatory pathways

**Figure 2.**
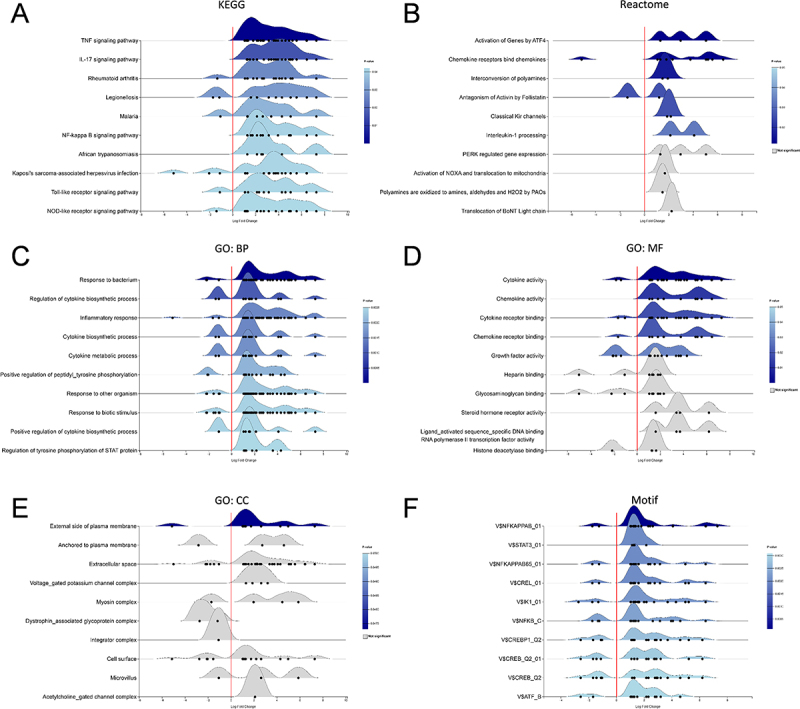
Functional enrichment analysis of differentially expressed genes following RS218 infection.

Functional enrichment analysis revealed that RS218 infection triggered comprehensive inflammatory responses and pathological processes consistent with bacterial meningitis pathogenesis (Figure 2). KEGG pathway analysis demonstrated activation of classical inflammatory signaling cascades, including TNF signaling pathway, IL-17 signaling pathway, and NF-κB signaling pathway, along with pathogen recognition pathways such as Toll-like receptor and NOD-like receptor signaling pathways (Figure 2(A)). Reactome pathway analysis further confirmed inflammatory activation and revealed the IL-1 processing pathway (Figure 2(B)). The enrichment of NOD-like receptor signaling and IL-1 processing pathways suggests potential activation of inflammasome-mediated responses.

Ridgeline plots showing the top 10 enriched terms in each category based on DEGs from RS218 infection. The color intensity represents the statistical significance (*p* value) of each term, while the distribution shape reflects the directional regulation of genes enriched in each term (left side: downregulated; right side: upregulated). (A) KEGG pathway. (B) Reactome pathway. (C) Gene Ontology Biological Process (GO:BP). (D) Gene Ontology Molecular Function (GO:MF). (E) Gene Ontology Cellular Component (GO:CC). (F) Binding motif.

Gene Ontology analysis confirmed predominant enrichment of bacterial response, inflammatory response, and cytokine biosynthetic processes, with corresponding molecular functions including cytokine and chemokine activities (Figure 2(C,D)). Cellular component analysis revealed enrichment of extracellular space and plasma membrane-associated terms, consistent with inflammatory mediator secretion and membrane alterations (Figure 2(E)). Several potentially novel pathways emerged, including Activation of Genes by ATF4, Interconversion of polyamines, and Classical Kir channels (Figure 2(B)), suggesting additional mechanisms beyond classical inflammatory responses. Binding motif analysis revealed enrichment of key inflammatory regulatory elements, particularly NF-κB, STAT3, and CREB/ATF binding motifs, indicating coordinated transcriptional reprogramming driving the host response to RS218 infection (Figure 2(F)).

### Hub gene identification shows coordinated inflammatory activation and growth factor signaling suppression

**Figure 3.**
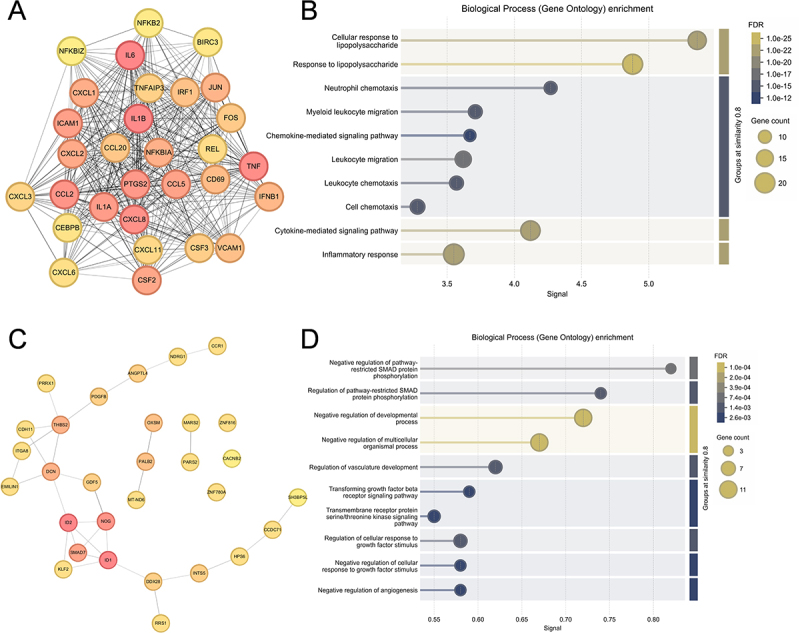
Protein-protein interaction network analysis and functional enrichment of hub genes. (A) Protein-protein interaction (PPI) network of upregulated DEGs with top 30 hub genes identified using the MCC (maximal clique centrality) algorithm in the cytoHubba plugin of Cytoscape. (B) Gene Ontology Biological Process enrichment analysis of the 30 hub genes. (C) PPI network of downregulated DEGs with top 30 hub genes identified using the MCC algorithm. (D) Gene Ontology Biological Process enrichment analysis of the 30 downregulated hub genes. For panels A and C, node color represents centrality. For panels B and D, dot size represents gene count, and color intensity indicates significance.

To identify key regulatory nodes in the gene expression network, we constructed a protein-protein interaction (PPI) network using upregulated DEGs and identified the top 30 hub genes using the cytoHubba plugin (Figure 3(A)). The network revealed highly interconnected inflammatory mediators, with central nodes including key cytokines (IL6, IL1A, IL1B, TNF, IFNB1), chemokines (CXCL1, CXCL2, CXCL3, CXCL6, CXCL8, CCL2, CCL5, CCL20), transcription factors (NFKB2, REL, JUN, FOS, IRF1), and inflammatory regulators (ICAM1, PTGS2, TNFAIP3). Functional enrichment analysis of these upregulated hub genes demonstrated their critical roles in bacterial infection responses, including cellular response to lipopolysaccharide, chemokine-mediated cell migration processes, and cytokine-mediated signaling pathways (Figure 3(B)).

Conversely, analysis of downregulated DEGs revealed a distinct network of hub genes involved in cellular growth, differentiation and developmental processes, including key regulators such as ID1, ID2, NOG, SMAD7, and DCN (Figure 3(C)). The downregulated hub genes were primarily enriched in growth factor signaling, particularly TGF-β/SMAD signaling (SMAD protein phosphorylation, transforming growth factor beta receptor signaling pathway) and developmental processes (developmental process regulation, angiogenesis) (Figure 3(D)). These findings suggest that RS218 infection not only activates inflammatory pathways but also suppresses growth factor-mediated regulatory mechanisms and developmental programs, potentially contributing to endothelial dysfunction and compromised blood-brain barrier (BBB) integrity.

### Proteomic enrichment analysis suggests cellular degradation and neural support dysfunction

**Figure 4.**
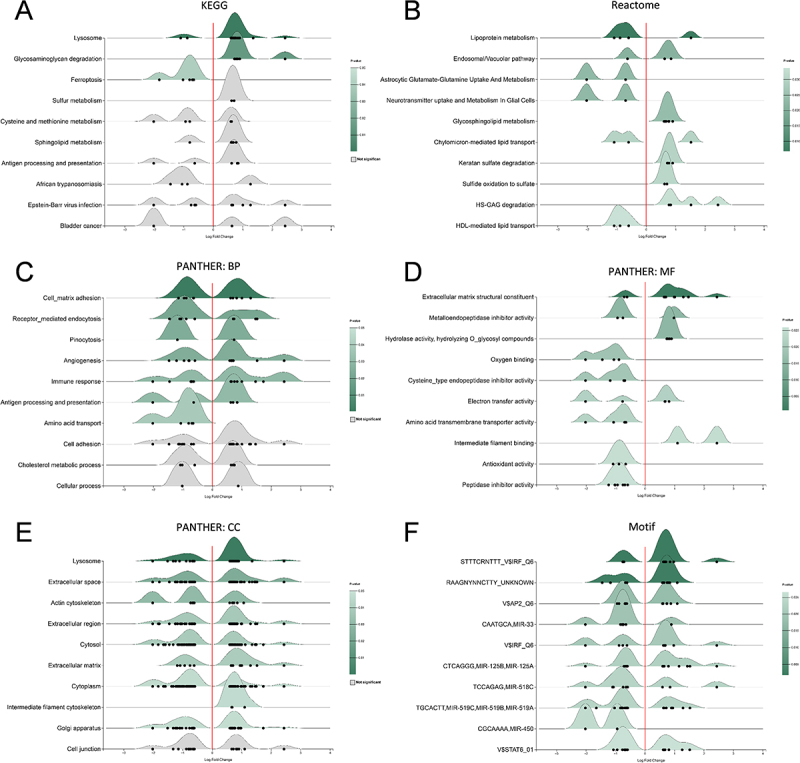
Functional enrichment analysis of differentially expressed proteins following RS218 infection. Ridgeline plots showing the top 10 enriched terms in each category based on DEPs from RS218 infection. The color intensity represents the statistical significance (*p* value) of each term, while the distribution shape reflects the directional regulation of proteins enriched in each term (left side: downregulated; right side: upregulated). (A) KEGG pathway. (B) Reactome pathway. (C) PANTHER Biological Process (PANTHER:BP). (D) PANTHER molecular function (PANTHER:MF). (E) PANTHER cellular component (PANTHER:CC). (F) binding motif.

For proteomic enrichment analysis, KEGG pathway analysis demonstrated activation of cellular degradation processes including lysosome and glycosaminoglycan degradation, while ferroptosis pathway was downregulated (Figure 4(A)). Reactome pathway analysis revealed activation of specific glycosaminoglycan degradation processes (Keratan sulfate degradation, HS-GAG degradation), providing more detailed specification of the KEGG glycosaminoglycan metabolism results. Neural metabolic processes including astrocytic glutamate-glutamine metabolism and glial neurotransmitter uptake were downregulated, suggesting compromised neural support functions (Figure 4(B)).

Biological process analysis showed enrichment of endocytosis and pinocytosis processes without clear directional patterns, along with immune response and antigen presentation pathways showing similar mixed regulation (Figure 4(C)). The only clear directional change was downregulation of amino acid transport, consistent with the compromised neural metabolic processes observed in Reactome analysis. Molecular function analysis revealed downregulation of oxygen-related activities (oxygen binding, antioxidant activity) (Figure 4(D)). Enhanced hydrolytic activity (hydrolase activity hydrolyzing O-glycosyl compounds) combined with decreased protease inhibitor activities (cysteine-type endopeptidase inhibitor, peptidase inhibitor, metalloendopeptidase inhibitor activity) indicated increased degradation processes, consistent with the glycosaminoglycan degradation pathways. Amino acid transmembrane transporter activity was downregulated, corresponding with the impaired neural metabolic processes (Figure 4(D)). Additionally, intermediate filament binding activity was upregulated, supported by cellular component analysis showing intermediate filament cytoskeleton, suggesting intermediate filament dynamics (Figure 4(E)). Lysosomal enrichment was also confirmed (Figure 4(E)), supporting the enhanced degradation pathways identified in previous analyses. Binding motif analysis revealed mixed regulation of immune-related transcription factors (IRF, STAT6) and clear downregulation of MIR-450, indicating potential microRNA-mediated regulatory mechanisms (Figure 4F).

### Hub protein analysis indicates complex immune and matrix remodeling patterns

**Figure 5.**
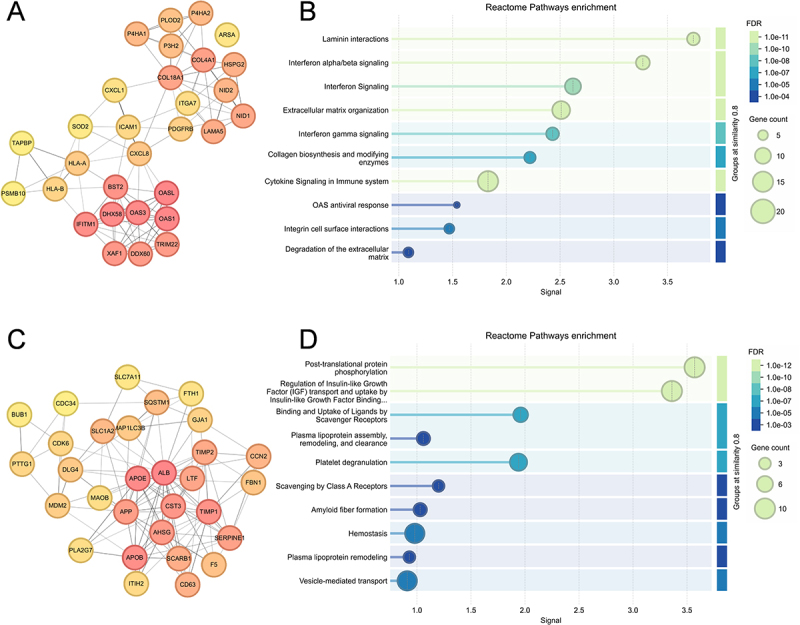
PPI network analysis and functional enrichment of hub proteins. (A) PPI network of upregulated DEPs with top 30 hub proteins identified using the MCC algorithm in the cytoHubba plugin of Cytoscape. (B) Reactome pathway enrichment analysis of the 30 upregulated hub proteins. (C) PPI network of downregulated DEPs with top 30 hub proteins identified using the MCC algorithm. (D) Reactome pathway enrichment analysis of the 30 downregulated hub proteins. For panels A and C, node color represents centrality. For panels B and D, dot size represents gene count, and color intensity indicates significance.

The upregulated protein network revealed antiviral/interferon response proteins (OAS1, OAS3, DDX60, DHX58, BST2, IFITM1, TRIM22, XAF1), extracellular matrix remodeling proteins including collagen components (COL4A1, COL18A1) and modifying enzymes (P4HA1, P4HA2, PLOD2), and antigen presentation and immune activation proteins (HLA-A, HLA-B, TAPBP, PSMB10, ICAM1, CXCL1, CXCL8) (Figure 5(A)). Functional enrichment analysis confirmed the functions of these hub proteins in interferon signaling and extracellular matrix organization (Figure 5(B)).

Conversely, the downregulated protein network displayed proteins involved in platelet function (F5, SERPINE1, PLA2G7, CD63), antimicrobial defense (LTF), scavenger receptor-mediated clearance (SCARB1), cell cycle regulation (BUB1, CDK6, PTTG1, CDC34, MDM2), and extracellular matrix/junction integrity (TIMP1, TIMP2, FBN1, CCN2, GJA1) (Figure 5(C)). Consistently, these hub proteins were enriched in vascular function regulation (platelet degranulation, hemostasis), scavenger receptor functions (binding and uptake of ligands, scavenging by Class A receptors), plasma lipoprotein metabolism, growth factor regulation (IGF transport), post-translational protein phosphorylation, and vesicle-mediated transport (Figure 5(D)). These findings indicate that RS218 infection reshapes endothelial immunity toward inflammatory responses while suppressing bacterial clearance pathways, accompanied by coordinated matrix remodeling.

### Integrative analysis depicts coordinated transcriptomic and proteomic activation

**Figure 6.**
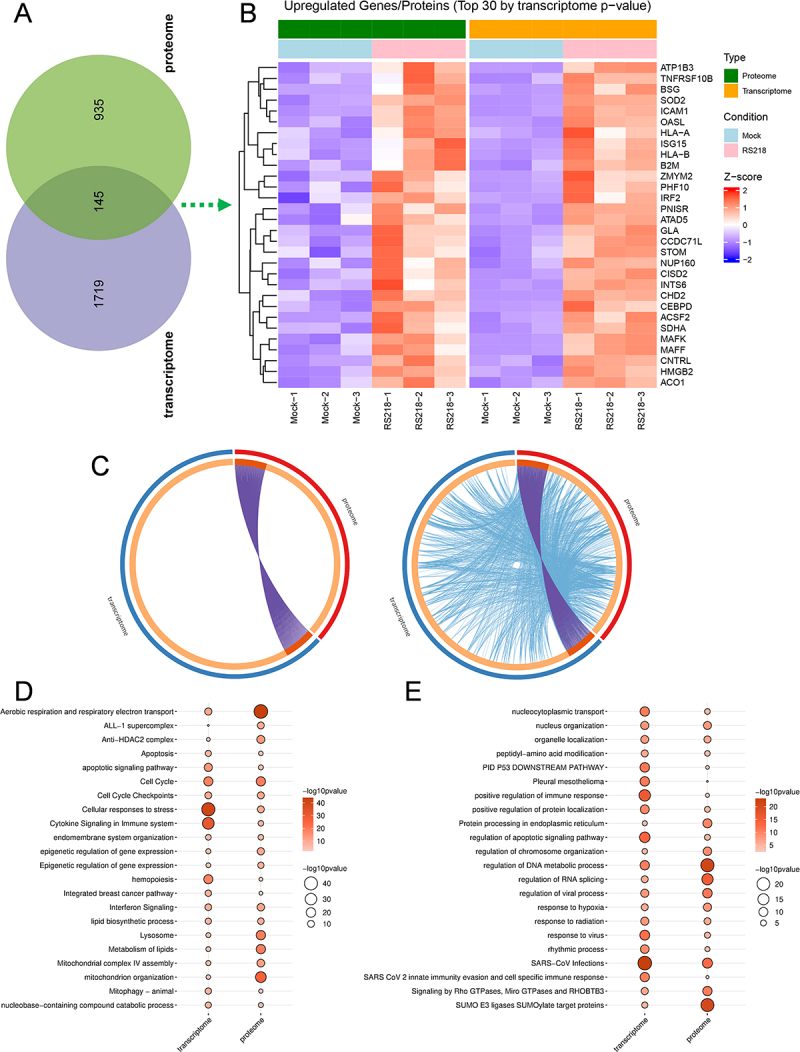
Multi-omics analysis of concordant upregulation patterns. (A) Venn diagram showing overlap between upregulated DEGs (padj < 0.05) and DEPs (*p* value < 0.05). (B) Heatmap displaying abundance patterns of the top 30 genes (selected by transcriptomic padj values) among the 145 commonly upregulated genes/proteins across both transcriptomic and proteomic datasets. Color intensity represents z-score normalized expression levels. (C) circos plots showing gene-level overlap (left) and functional pathway-level connections (right) between upregulated DEGs and DEPs. Purple curves represent identical genes; blue curves connect different genes belonging to the same enriched ontology terms. (D-E) shared functional terms enriched in both upregulated transcriptomic and proteomic datasets. Dot size and color intensity represent significance level.

To identify genes with concordant regulation across transcriptomics and proteomics, we performed integrative analysis using padj < 0.05 for DEGs and *p* value < 0.05 for DEPs to capture broader regulatory patterns. The analysis revealed 145 genes that were consistently upregulated in both datasets from transcriptomic (1864 genes total) and proteomic (1080 proteins total) responses (Figure 6(A)). The most significant genes included antigen presentation components (HLA-A, HLA-B, B2M), interferon-stimulated genes (ISG15, OASL, IRF2), and several other immune regulatory proteins (CEBPD, ICAM1, BSG, HMGB2, SOD2, TNFRSF10B) (Figure 6(B)). Additional shared genes encompassed numerous immune mediators, including chemokines (CXCL1, CXCL8), immune regulatory and signaling molecules (HERC5, STAT2, IRF9, RFX1, LGALS1, BCAP31), cell surface proteins (CD9, CD46, CD109, NECTIN2), and mitochondrial respiratory chain complex IV components (COX4I1, COX5B, COX6A1, COX6B1, COX7A2, COX7C) (Table S3).

Further integrative functional analysis revealed that while gene-level overlap between transcriptomics and proteomics was limited, extensive pathway-level convergence existed, with numerous distinct genes from both datasets participating in shared biological processes and signaling pathways (Figure 6(C)). This suggests coordinated functional responses despite differences in individual gene/protein regulation between transcriptomics and proteomics. The integrative analysis demonstrated extensive functional overlap between upregulated transcriptomics and proteomics, with shared enrichment in aerobic respiration and respiratory electron transport, cellular stress responses and cell death regulation (apoptosis, cellular responses to stress), epigenetic regulation (chromosome organization), immune system activation including cytokine signaling and interferon signaling, lysosomal pathways, and hematopoietic processes (Figure 6(D,E)). Some functional terms were enriched only in upregulated DEGs but not in DEPs, including key innate immune signaling pathways such as response to LPS, NF-κB, TNF, TLR3, and MAPK signaling (Table S4). Conversely, some terms were enriched only in upregulated DEPs, and they were mainly related to metabolism and degradation, antigen processing and presentation, DNA repair, and collagen formation (Table S4).

### Coordinated downregulation of cellular maintenance and barrier integrity functions

**Figure 7.**
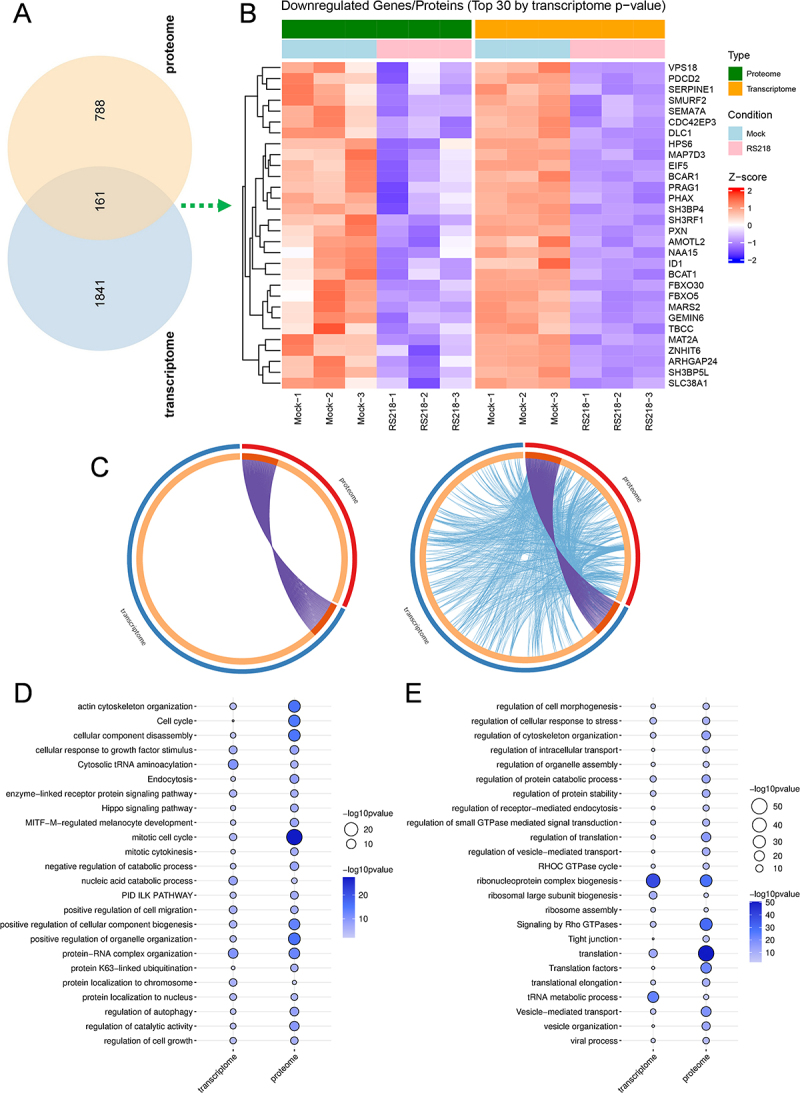
Multi-omics analysis of concordant downregulation patterns. (A) Venn diagram showing overlap between downregulated DEGs (padj < 0.05) and DEPs (*p* value < 0.05). (B) Heatmap displaying abundance patterns of the top 30 genes (selected by transcriptomic padj values) among the 161 commonly downregulated genes/proteins across both transcriptome and proteome datasets. Color intensity represents z-score normalized expression levels. (C) circos plots showing gene-level overlap (left) and functional pathway-level connections (right) between downregulated DEGs and DEPs. Purple curves represent identical genes; blue curves connect different genes belonging to the same enriched ontology terms. (D-E) shared functional terms enriched in both downregulated transcriptomic and proteomic datasets. Dot size and color intensity represent significance level.

The integrative analysis of downregulated genes revealed 161 genes that were consistently downregulated in both transcriptomics and proteomics, with 1841 genes unique to transcriptome and 788 proteins unique to proteome (Figure 7(A)). The most significant genes included cytoskeletal regulators (SEMA7A, CDC42EP3, DLC1, PXN, ARHGAP24, MAP7D3, TBCC), neural function proteins (SLC38A1), endothelial regulators (AMOTL2), and translation regulators (EIF5, ZNHIT6, MARS2) (Figure 7(B)).

Additional shared downregulated genes complemented the top 30 findings and revealed broad suppression of cellular functions essential for endothelial barrier integrity and homeostasis (Table S5). These included extensive downregulation of translation machinery (AARS1, AARSD1, EIF2B2, EIF3J, EPRS1, GARS1, MARS1, TARS1, YARS1), cytoskeletal organization components (ACTG1, ARAP1, ARHGAP1, ARHGEF18, CALD1, MACF1, MAP1B, VCL, ZYX), neural function proteins (AP3M2, DLGAP4, NEPRO), and oxidative stress response proteins (GLRX2, GSR, HMOX1, TXNRD1). Critically, the tight junction protein TJP1 (ZO-1) was downregulated in both datasets, providing direct evidence for compromised BBB integrity (Table S5).

Integrative analysis of downregulated genes showed similar patterns to upregulated datasets, with limited shared genes but extensive shared signaling pathways between transcriptomics and proteomics (Figure 7(C)). Pathway analysis of downregulated genes showed functional overlap in essential cellular maintenance processes (Figure 7(D,E)). Key enriched pathways included cytoskeletal organization (actin cytoskeleton organization, regulation of cytoskeleton organization, Rho GTPase signaling), translational regulation (cytosolic tRNA aminoacylation, translation, ribosome assembly, ribosomal large subunit biogenesis), protein stability regulation (protein K63-linked ubiquitination, regulation of protein catabolic process), and tight junction integrity (tight junction). The extensive downregulation of translational machinery and protein stability pathways provides mechanistic insight into the observed differences between transcriptomic and proteomic responses, while the suppression of tight junction and cytoskeletal pathways directly supports compromised BBB integrity.

Additionally, four pathways were enriched only for downregulated DEGs, including maturation of 5.8S rRNA, stem cell maintenance, DNA metabolic regulation, and mitochondrial translation. Conversely, pathways enriched only for downregulated DEPs pointed out several key terms such as TRIF (TICAM1)-mediated TLR4 signaling, ferroptosis, and autophagy (Table S6). Cell cycle pathways were enriched as both upregulated and downregulated terms (Figure 6(D), 7(D)), suggesting bidirectional regulation. Many other terms showed similar patterns of enrichment in both directions (Table S7), such as chromatin remodeling, response to oxidative stress, and VEGFA-VEGFR2 signaling.

### Discordant transcriptomic and proteomic regulation reveals complex post-transcriptional control mechanisms

**Figure 8.**
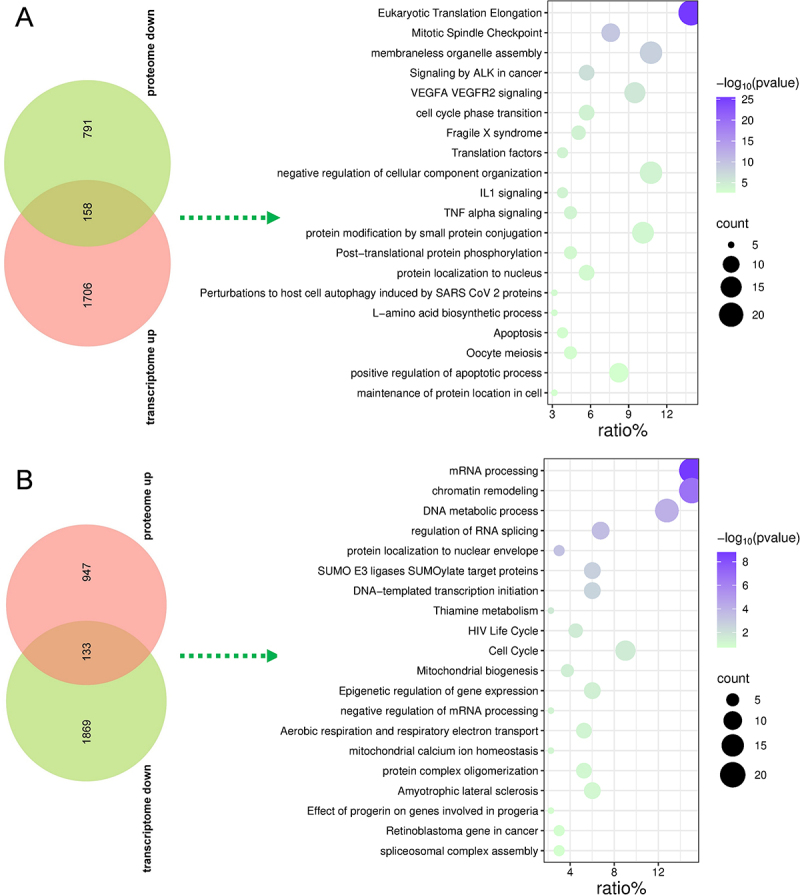
Analysis of genes with discordant regulation between transcriptomic and proteomic levels. (A) Venn diagram and functional enrichment analysis of genes upregulated in transcriptome but downregulated in proteome. (B) Venn diagram and functional enrichment analysis of genes downregulated in transcriptome but upregulated in proteome. Dot size represents gene count and color intensity indicates significance level.

Analysis of genes with discordant regulation between transcriptomic and proteomic levels revealed 158 genes that were upregulated in transcriptome but downregulated in proteome, and 133 genes showing the opposite pattern (Figure 8(A,B); Table S8). Genes upregulated at mRNA level but downregulated at protein level were enriched in translation processes, cell cycle regulation, VEGFA-VEGFR2 signaling, protein homeostasis and modification pathways, inflammatory signaling (IL1 and TNF alpha signaling), and apoptosis, suggesting active transcriptional responses that may be counteracted by protein degradation or translational inhibition during RS218 infection (Figure 8(A)). Conversely, genes downregulated at mRNA level but upregulated at protein level were enriched in DNA and RNA processing, cell cycle regulation, epigenetic processes, and mitochondrial function, indicating potential post-transcriptional stabilization or accumulation of proteins despite reduced transcription (Figure 8(B)). These findings highlight the complex regulatory mechanisms operating during bacterial infection, where transcriptional and post-transcriptional controls can act in opposing directions to fine-tune cellular responses.

### RS218 infection induces cell death and tight junction disruption in vitro

**Figure 9.**
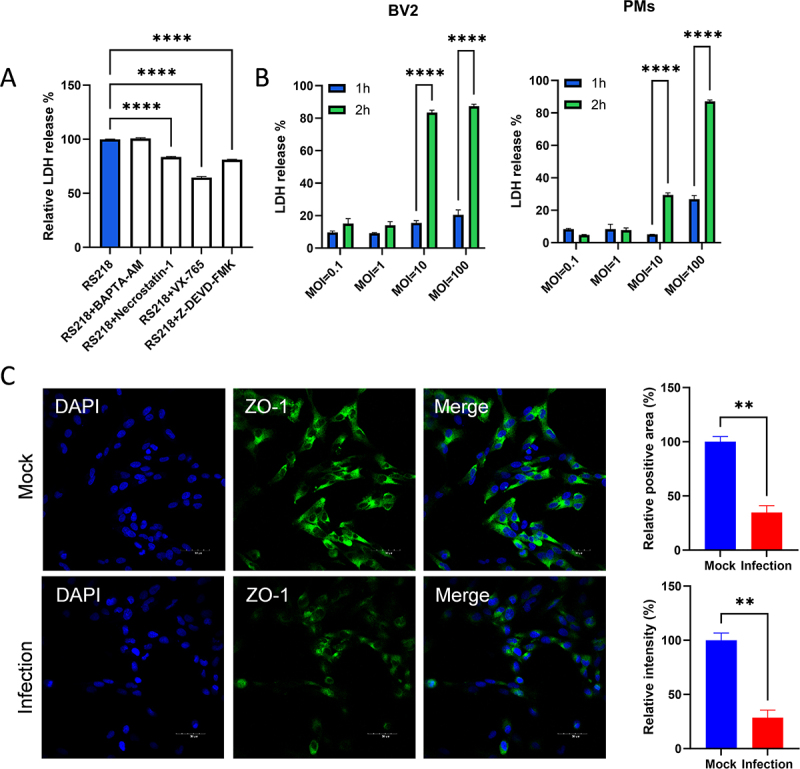
RS218 infection induces cell death and tight junction disruption *in vitro*. (A) Relative LDH release in RS218-infected hCMEC/D3 cells pretreated with calcium chelator BAPTA-AM (10 μM), necroptosis inhibitor Necrostatin-1 (10 μM), caspase-1 inhibitor VX-765 (10 μM), or caspase-3 inhibitor Z-DEVD-FMK (20 μM). (B) LDH release in BV2 cells and murine peritoneal macrophages (PMs) infected with RS218 at MOI = 0.1, 1, 10, and 100 for 1 h and 2 h. (C) Immunofluorescence staining of tight junction protein ZO-1 in hCMEC/D3 cells following RS218 infection. Representative images show DAPI (blue), ZO-1 (green), and merged channels in control and infected cells. Scale bars, 50 μm. Bar graphs show ZO-1 positive area and fluorescence intensity in infected cells relative to controls (*n* = 3).

To validate cell death pathways identified in the omics analysis, LDH release assays were performed in RS218-infected hCMEC/D3 cells treated with calcium chelator BAPTA-AM, necroptosis inhibitor Necrostatin-1, caspase-1 inhibitor VX-765, and caspase-3 inhibitor Z-DEVD-FMK. Necrostatin-1, VX-765, and Z-DEVD-FMK significantly reduced LDH release, while BAPTA-AM had no significant effect (Figure 9(A)). RS218 also induced significant LDH release in the murine microglial cell line BV2 and murine peritoneal macrophages (PMs) in a dose- and time-dependent manner (Figure 9(B)). Immunofluorescence staining of hCMEC/D3 cells confirmed that RS218 infection significantly disrupted tight junction integrity, with reduced ZO-1 positive area and fluorescence intensity (Figure 9(C)). This validated the integrated analysis showing TJP1 (encoding ZO-1) among concordantly downregulated genes, providing molecular evidence for compromised barrier function during meningitis-associated ExPEC infection.

### RS218 infection induces systemic dysregulation, blood-brain barrier disruption, and brain inflammation in vivo

**Figure 10.**
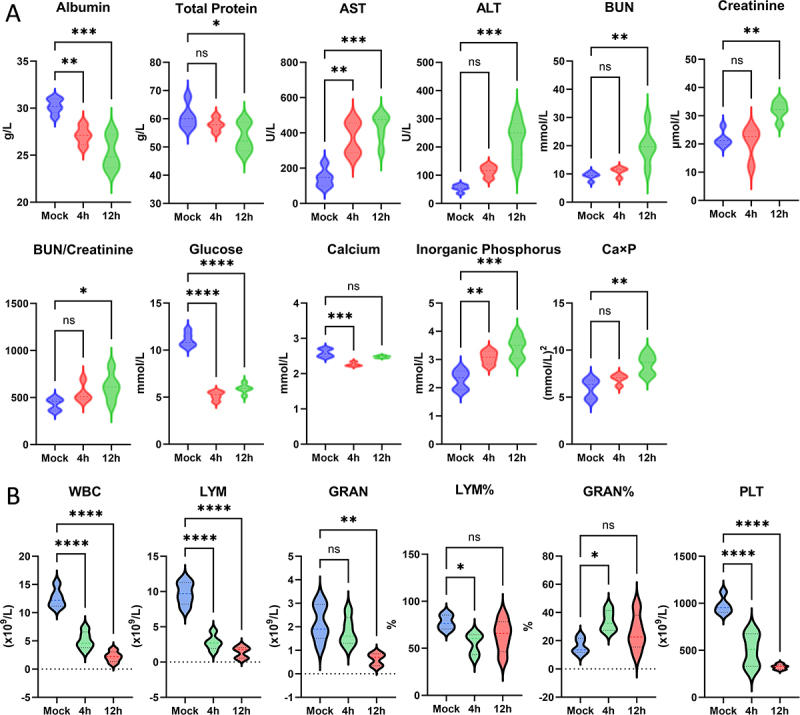
RS218 infection induces systemic biochemical and hematological changes in mice. (A) blood biochemical parameters measured in mock-infected controls and in mice at 4 and 12 hours post-infection. Parameters include albumin, total protein, aspartate aminotransferase (AST), alanine aminotransferase (ALT), blood urea nitrogen (BUN), creatinine, BUN/creatinine ratio, glucose, calcium, inorganic phosphorus, and calcium-phosphorus product (Ca×P). (B) hematological parameters including white blood cell count (WBC), lymphocyte count (LYM), granulocyte count (GRAN), lymphocyte percentage (LYM%), granulocyte percentage (GRAN%), and platelet count (PLT).

Systemic RS218 infection induced significant biochemical and hematological changes in mice (Figure 10, Figure S2). Blood biochemical analysis revealed decreased albumin, total protein, glucose, and calcium levels, along with increases in AST, ALT, BUN, creatinine, BUN/creatinine ratios, inorganic phosphorus, and calcium-phosphorus product (Figure 10(A)), indicating hepatic and renal injury and widespread metabolic dysregulation. Additional biochemical parameters including globulin, albumin/globulin ratio, total bilirubin, amylase, creatine kinase, triglyceride, and AST/ALT ratio showed no significant changes following infection (Figure S2A). Hematological analysis showed profound leukopenia with decreased white blood cell, lymphocyte, and granulocyte counts, along with decreased lymphocyte percentage and increased granulocyte percentage (Figure 10(B)). Platelet counts were reduced, a hallmark feature of severe systemic bacterial infection that may indicate consumption coagulopathy (Figure 10(B)). Additional hematological parameters including red blood cell indices and mid-sized cell parameters showed no significant changes following infection (Figure S2B). Among platelet-related parameters, MPV, PDW, P_LCR, and P_LCC remained unchanged, while PCT was significantly decreased (Figure S2B).

**Figure 11.**
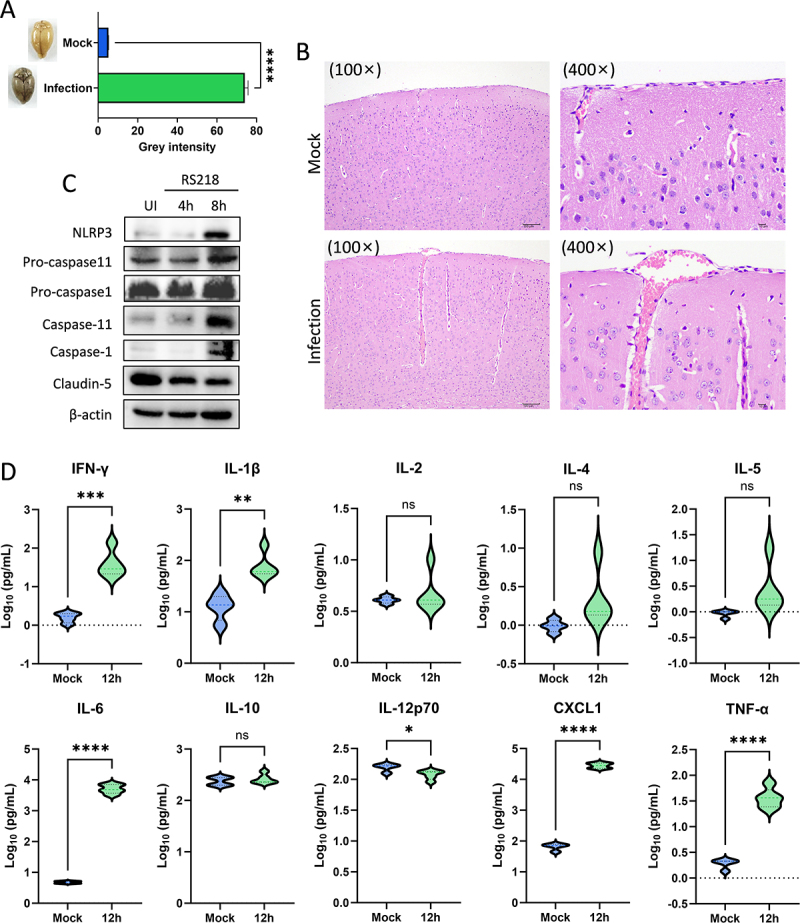
RS218 infection disrupts blood-brain barrier integrity and induces neuroinflammation *in vivo*. (A) Evans Blue extravasation in mouse brain tissue following RS218 infection. Representative images and quantification of gray intensity are shown. (B) Hematoxylin and eosin (H&E) staining of brain tissue sections from mock and RS218-infected mice at 100× and 400× magnification. (C) western blot analysis of brain tissue from mock and RS218-infected mice detecting NLRP3, pro-caspase-1, caspase-1, pro-caspase-11, caspase-11, Claudin-5, and β-actin. (D) cytokine levels in brain tissue from mock-infected controls and from mice at 12 hours post-infection. Ten cytokines were measured: interferon-γ (IFN-γ), interleukin-1β (IL-1β), IL-2, IL-4, IL-5, IL-6, IL-10, IL-12p70, C-X-C motif chemokine ligand 1 (CXCL1), and tumor necrosis factor-α (TNF-α).

Brain tissue analysis further confirmed BBB disruption and neuroinflammation *in vivo*. Evans Blue extravasation was significantly increased in infected mouse brains, demonstrating functional BBB permeability disruption (Figure 11(A)). Histological examination revealed capillary congestion and dilation in brain tissue from infected mice (Figure 11(B)). Western blot analysis of brain tissue demonstrated NLRP3 inflammasome activation, caspase-1/11-dependent pyroptosis signaling, and degradation of the tight junction protein Claudin-5 (Figure 11(C)). Cytokine analysis of brain tissue confirmed significant neuroinflammation with elevated IFN-γ, IL-1β, IL-6, CXCL1, and TNF-α, while IL-12p70 was decreased and IL-2, IL-4, IL-5, and IL-10 remained unchanged (Figure 11(D)). These findings validated the BBB disruption and inflammatory activation identified in the omics analysis.

## Discussion

This integrated transcriptomic and proteomic analysis provides a comprehensive molecular landscape of host responses during meningitis-associated ExPEC infection of brain endothelial cells. The study reveals coordinated inflammatory activation, direct evidence for blood-brain barrier (BBB) disruption, and widespread metabolic dysregulation, with experimental validation confirming these findings in both cellular and animal models.

RS218 infection triggered robust inflammatory activation across multiple molecular levels. Transcriptomic analysis demonstrated strong upregulation of inflammatory mediators, including cytokines, chemokines, and transcription factors, with activation of classical inflammatory signaling cascades such as Toll-like receptor, NOD-like receptor, NF-κB, TNF, and IL-1 signaling pathways. Proteomic analysis revealed upregulation of interferon-stimulated genes and antigen presentation components. Integrative analysis identified 145 genes concordantly upregulated at both transcriptomic and proteomic levels, containing extensive immune-related genes involved in antigen presentation, interferon responses, cytokine signaling, and immune regulation, demonstrating coordinated inflammatory and immune activation. Although the number of overlapping genes was relatively limited, the transcriptomic and proteomic datasets showed substantial functional integration at the pathway level, with shared activation of immune system responses including interferon signaling and cytokine signaling.

Interestingly, the discordant regulation analysi s revealed that genes upregulated at the transcriptional level but downregulated at the protein level were enriched in inflammatory signaling pathways, including IL-1 and TNF-α signaling. This discordance suggests that while transcriptional programs strongly activate inflammatory responses, multiple post-transcriptional [36,37] and post-translational [38] regulatory mechanisms, such as mRNA degradation, translational suppression, and protein degradation, may serve to limit excessive inflammatory protein accumulation, representing a potential protective mechanism to prevent overwhelming inflammatory damage to the endothelial barrier.

Despite the post-transcriptional or post-translational regulation, overall inflammatory responses remained robust, and the functional significance of these inflammatory mediators in BBB disruption is well established. Specifically, several key inflammatory cytokines including IL-1β, IL-6, and TNF-α have been previously demonstrated to be principally responsible for BBB breakdown [10]. These findings establish a strong link between the observed inflammatory activation and BBB disruption during meningitis-associated ExPEC infection. Animal experiments validated these findings, with peripheral blood showing significant alterations in immune cell populations and brain tissue demonstrating elevated levels of multiple pro-inflammatory cytokines.

A critical finding was the identification of direct molecular evidence for BBB integrity compromise. TJP1, encoding the tight junction protein ZO-1, was concordantly downregulated in both transcriptome and proteome, providing direct evidence for tight junction disruption. ZO-1 is a crucial scaffolding protein that connects transmembrane tight junction proteins to the actin cytoskeleton and regulates barrier permeability [39]. Immunofluorescence validation confirmed significant reduction in ZO-1 positive area and fluorescence intensity in infected cells, supporting the omics findings. *In vivo*, Evans Blue extravasation demonstrated functional BBB permeability disruption, and western blot analysis of brain tissue further confirmed degradation of the tight junction protein Claudin-5, collectively supporting the omics findings of tight junction disruption. Notably, TJP2 was differentially expressed only at the protein level, suggesting distinct degradation mechanisms for different tight junction components and highlighting the importance of multi-omics approaches in capturing the full spectrum of molecular changes. Beyond tight junction proteins, cytoskeletal reorganization was evident, with upregulation of intermediate filament cytoskeleton and downregulation of multiple cytoskeletal regulators and suppression of actin cytoskeleton organization and Rho GTPase signaling pathways [40]. The downregulation of GJA1, which encodes Connexin 43, a gap junction protein previously shown to be important for intercellular communication, suggests that gap junction-mediated communication may also be compromised in endothelial cells during bacterial meningitis, which may further contribute to BBB dysfunction [41].

An important mechanism contributing to BBB disruption was revealed through the activation of glycosaminoglycan degradation pathways in the proteomic analysis. The endothelial glycocalyx serves as the initial gatekeeper for barrier functions at the luminal side of the BBB and is comprised of proteoglycans, glycosaminoglycans, glycoproteins and associated plasma proteins [42]. The proteomic analysis showed activation of specific glycosaminoglycan degradation processes, including keratan sulfate degradation and HS-GAG degradation. Additionally, enhanced hydrolase activity hydrolyzing O-glycosyl compounds, combined with decreased protease inhibitor activities, indicates increased degradation of both glycosaminoglycan chains and protein components. This may accelerate the turnover of glycocalyx and barrier proteins, thereby compromising BBB structural integrity. These findings complement previous studies demonstrating that meningitis-associated ExPEC disrupts BBB integrity through multiple mechanisms, including upregulation of vascularization factors such as VEGFA, PDGF-B, and ANGPTL4 [11]. The current study provides a more comprehensive view of the coordinated molecular mechanisms underlying BBB compromise during ExPEC infection.

RS218 infection induced cell death in brain endothelial cells, as demonstrated by LDH release assays. Treatment with necroptosis inhibitor Necrostatin-1, caspase-1 inhibitor VX-765, and caspase-3 inhibitor Z-DEVD-FMK significantly reduced LDH release in infected hCMEC/D3 cells, while calcium chelator BAPTA-AM had no significant effect, suggesting the involvement of multiple cell death pathways. RS218 also induced cell death in murine microglial BV2 cells and murine peritoneal macrophages, indicating that cell death is not restricted to brain endothelial cells during infection. Consistent with these *in vitro* findings, western blot analysis of brain tissue confirmed NLRP3 inflammasome activation and caspase-1/11-dependent pyroptosis signaling *in vivo*, highlighting cell death as a key mechanism contributing to BBB disruption during meningitis-associated ExPEC infection.

Metabolic dysregulation and cellular homeostasis disruption emerged as prominent features of the host response. The downregulation of neural metabolic processes, including astrocytic glutamate-glutamine metabolism and amino acid transport, reflects compromised neural support functions [43,44]. This impairment of metabolic support may contribute to neurological complications observed in bacterial meningitis beyond direct inflammatory damage [45]. A striking finding was the downregulation of translational machinery, encompassing multiple aminoacyl-tRNA synthetases and translation initiation factors. This translational suppression likely represents a cellular stress response to limit protein synthesis during infection [46] and provides a mechanistic explanation for the observed discordance between transcriptomic and proteomic responses. Consistent with impaired protein synthesis, the animal model demonstrated decreased levels of blood proteins, further supporting disruption of protein homeostasis during ExPEC infection. The animal model also demonstrated severe systemic metabolic disruption, with decreased levels of glucose and calcium, along with elevated markers of hepatic and renal injury. These findings indicate that ExPEC infection causes widespread organ dysfunction beyond the CNS, which may contribute to the overall disease severity and complicate therapeutic interventions.

The downregulation of growth factor signaling pathways, particularly TGF-β/SMAD signaling, represents another important finding with implications for BBB integrity. Hub gene analysis of downregulated transcripts identified key regulators of growth factor responses and TGF-β/SMAD signaling. This finding is noteworthy given that TGF-β1-mediated signaling has been previously demonstrated to enhance endothelial tight junction protein expression and maintain BBB integrity through astrocyte-endothelial communication [47], and that ExPEC α-hemolysin disrupts this protective signaling by inhibiting TGFBRII and Gli1/2-mediated pathways [13]. The observed downregulation of TGF-β/SMAD signaling components in infected endothelial cells suggests that loss of this protective pathway may contribute to BBB compromise. Furthermore, the downregulation of developmental processes may further impair the ability of the BBB to repair and regenerate following infection-induced damage.

This study has several limitations that should be acknowledged. The different infection durations for transcriptomic (2 hours) and proteomic (3 hours) analyses may introduce temporal variability, though this design was chosen to capture robust protein-level changes that typically require longer timeframes than transcriptional responses. The study focused on a single ExPEC strain and an immortalized cell line, and validation with additional strains and primary brain endothelial cells would strengthen the generalizability of the findings. Future functional studies combining genetic manipulation of identified targets with BBB permeability assays would establish causality and provide stronger evidence for therapeutic target validation.

The identification of inflammatory activation, tight junction degradation, and metabolic dysregulation provides multiple potential therapeutic targets. The extensive translational suppression and post-transcriptional regulation mechanisms open new possibilities for therapeutic intervention at the translational level.

In conclusion, this integrated transcriptomic and proteomic analysis reveals coordinated inflammatory activation, multilayered barrier disruption mechanisms, widespread metabolic dysregulation, and extensive post-transcriptional regulation during meningitis-associated ExPEC infection of brain endothelial cells. The validation of inflammatory activation and barrier disruption in cellular and animal models supports the reliability of the omics findings. These results advance our understanding of the host-pathogen interactions underlying ExPEC-induced BBB compromise and identify multiple potential therapeutic targets.

## Ethics statement

Ethical approval was not required for studies involving human-derived cell lines in accordance with local legislation and institutional requirements, as only commercially available established cell lines were used. The animal study was approved by the Institutional Animal Care and Use Committee (IACUC) of Southwest University, Chongqing, China (IACUC-20221022-08). The study was conducted in accordance with the local legislation and institutional requirements. All animal experiments in this study were in accordance with the ARRIVE guidelines.

## Data Availability

The RNA-seq data for this study have been deposited in the European Nucleotide Archive (ENA) at EMBL-EBI under accession number PRJEB94539. The mass spectrometry proteomics data have been deposited to the ProteomeXchange Consortium via the PRIDE [32] partner repository with the dataset identifier PXD066627. Raw and supplementary data for this study are available at Figshare (DOI: 10.6084/m9.figshare.30338854).

## Supplementary Information

Supplemental data for this article can be accessed online at https://doi.org/10.6084/m9.figshare.30338854
